# Laurolitsine ameliorates insulin resistance, ovarian dysfunction and gut microbiota dysbiosis in DHEA plus HFD-induced PCOS mice

**DOI:** 10.3389/fendo.2025.1517934

**Published:** 2025-09-08

**Authors:** Yong Zhang, Mimi Chen, Zhi Zhou, Yichen Guo, Linlin Zheng, Yansu Yu, Jialu Liu, Yuanyuan Zhou, Hui Lu, Si Yao, Xiaopo Zhang, Ning Ma, Weiying Lu

**Affiliations:** ^1^ Key Laboratory of Tropical Translational Medicine of Ministry of Education, School of Basic Medicine and Life Sciences, Hainan Academy of Medical Sciences, Hainan Medical University, Haikou, China; ^2^ Hainan Academy of Medical Sciences, Hainan Medical University, Haikou, China; ^3^ Reproductive Medical Center, Hainan Women and Children’s Medical Center, Haikou, China; ^4^ Haikou Key Laboratory of Li Nationality Medicine, Hainan Key Laboratory for Research and Development of Tropical Herbs, Engineering Research Center of Tropical Medicine Innovation and Transformation of Ministry of Education and International Joint Research Center of Human-Machine Intelligent Collaborative for Tumor Precision Diagnosis and Treatment of Hainan Province, School of Pharmacy, Hainan Academy of Medical Sciences, Hainan Medical University, Haikou, China

**Keywords:** laurolitsine, PCOS, insulin resistance, hormonal imbalance, ovarian dysfunction, gut microbiota

## Abstract

**Background:**

Polycystic ovary syndrome (PCOS) is a complex endocrine reproductive disorder that affects 10%-13% of women worldwide, characterized by hyperandrogenemia, ovulatory dysfunction, and polycystic ovary formation. Currently, there are no effective specific treatments for PCOS. Therefore, identifying safe and effective therapeutic drugs for PCOS is clinically important.

**Methods:**

In this study, a PCOS mouse model was induced using dehydroepiandrosterone (DHEA) plus high-fat diet (HFD) to investigate the therapeutic effects of laurolitsine (LL). The efficacy of LL was evaluated by estrous cycle, glucose tolerance test (OGTT), insulin tolerance test (ITT), and serum biochemical markers. Histopathological analysis of ovarian, gonadal fat, and liver tissues was performed using hematoxylin and eosin (H&E) staining. Furthermore, RNA-seq analysis and 16S rRNA sequencing were performed to explore the potential mechanisms underlying LL’s effects on PCOS mice.

**Results:**

LL exhibited therapeutic effects in PCOS mice. LL improved lipid metabolism, glucose tolerance, insulin resistance, hormonal imbalance, and ovarian dysfunction in PCOS mice. RNA-seq analysis revealed that LL may improve PCOS by modulating key metabolic processes, including hormone response, fatty acid metabolism, and lipid metabolism in the ovaries. Additionally, LL significantly modulated the gut microbiota composition in PCOS mice, particularly reducing the abundance of *Proteobacteria* and *Lactobacillus johnsonii*, while increasing the abundance of *Akkermansia muciniphila*.

**Conclusion:**

LL is a promising and novel therapeutic agent for PCOS, as it ameliorates insulin resistance, ovarian dysfunction, and gut microbiota.

## 1 Introduction

Polycystic ovary syndrome (PCOS) is a complex endocrine reproductive disorder with a global prevalence of 10%-13% among women of reproductive age (1–4). It is characterized by hyperandrogenemia, ovulatory dysfunction and polycystic ovary morphology, often accompanied by metabolic disorders such as insulin resistance, obesity, and metabolism-associated fatty liver disease (MAFLD) (3–6).

Hyperandrogenism is one of the core pathological features of PCOS and a major factor in its development. In PCOS patients, excessive secretion of luteinizing hormone (LH) suppresses the secretion of follicle-stimulating hormone (FSH), disrupting endocrine balance (7). This hormonal imbalance not only affects ovarian function but also further exacerbates PCOS symptoms (8). Therefore, regulating hormone levels is crucial for the effective treatment of PCOS. Ovarian dysfunction, one of the core clinical manifestations of PCOS, typically presents as ovulatory disorders and polycystic ovary morphology. This dysfunction is closely associated with hyperandrogenemia, chronic anovulation, and abnormal follicular development (3, 9). Therefore, regulating ovarian function is critical for improving PCOS. Gut microbiota dysbiosis plays a crucial role in the pathogenesis of PCOS. Research has revealed significant differences in the gut microbiota composition between PCOS patients and healthy individuals, with PCOS patients exhibiting reduced microbial diversity and an overgrowth of certain pathogenic bacteria (10, 11). These microbial alterations are closely linked to insulin resistance and inflammatory responses, which further exacerbate the progression of PCOS (12). Therefore, modulating the gut microbiota has emerged as a promising therapeutic target for improving metabolic and reproductive dysfunctions in PCOS patients (13, 14).

Traditional treatments for PCOS, such as oral contraceptives and metformin, are effective at controlling symptoms but often come with side effects that limit their long-term clinical application. Oral contraceptives may increase the risk of thromboembolic events, and mood changes, while metformin commonly causes gastrointestinal disturbances such as nausea, diarrhea, and abdominal discomfort (15, 16). Consequently, exploring novel, safe, and effective therapeutic strategies for PCOS is of great clinical significance. Laurolitsine (LL), a natural product derived from the *Litsea glutinosa* (Lour.), has been used in traditional Chinese medicine for the treatment of metabolic disorders including diabetes (17, 18). Our previous research has demonstrated that LL exerts therapeutic effects on type 2 diabetes mellitus (T2DM) by improving glucose and lipid metabolism and modulating gut microbiota (19). In this study, we aimed to investigate the effects of LL in dehydroepiandrosterone (DHEA) combinate high-fat diet (HFD)-induced PCOS mice model to reveal its potential mechanisms.

## 2 Materials and methods

### 2.1 Reagents

Laurolitsine (PubChem CID: 22179, Catalog Number: BP0922, Purity ≥ 98%) was supplied by Desite (Chengdu, China). Dehydroepiandrosterone (DHEA) (PubChem CID: 5881, Catalog Number: GC11070, Purity ≥ 99%) was supplied by Glpbio (USA). Metformin (PubChem CID:4091, Catalog Number: S30880, Purity ≥ 98%) was supplied by Shyuanye (Shanghai, China). A high-fat diet (HFD, Catalog Number: D12492, 60 Kcal% Fat) was supplied by Future Biotech (Beijing, China). The triglyceride (TG, Cat Number: A110-1-1), cholesterol (CHO, Cat Number: A111-1-1), low density lipoprotein cholesterol (LDL-c, Cat Number: A113-1-1), high density lipoprotein cholesterol (HDL-c, Cat Number: A112-1-1), aspartate aminotransferase (AST, Cat Number: C010-2-1), and alanine aminotransferase (ALT, Cat Number: C009-2-1) assay kits were purchased from Njjcbio (Nanjing, China). The serum testosterone (T, Cat Number: E-OSEL-M0003), luteinizing hormone (LH, Cat Number: E-EL-M3053), estradiol (E2, Cat Number: E- E-OSEL-M0008), and follicle-stimulating hormone (FSH, Cat Number: E-EL-M0511) enzyme-linked immunosorbent assay (ELISA) kits were purchased from Elabscience (Wuhan, China).

### 2.2 Animal experiment

A total of 40 three-week-old female C57BL/6J mice were purchased from Gempharmatech Co., Ltd. (Jiangsu, China). The animal study was conducted according to the regulations of the National Institutes of Health (NIH), USA. The experimental protocol was approved by the Ethics Committee of Hainan Medical University (Approval No. SYXK-2017 0013). All mice were housed in standard cages under controlled environmental conditions (25°C, appropriate humidity, and a 12-h light/12-h dark cycle), with free access to food and water. Daily health monitoring was performed throughout the experimental period. After three days of acclimation, the mice were randomly divided into two groups: the normal group (n=8) and the PCOS model group (n=32). The normal group was fed a basic diet, whereas the model group was fed an HFD. From day 8 to day 28 (weeks 2 to 4), the model group received daily injections of DHEA (0.6 mg/kg/d) in combination with the HFD to induce PCOS. Meanwhile, the normal group continued on the basic diet and received vegetable oil injections as a control.

In the week 5, body weight, estrous cycle and oral glucose tolerance test (OGTT) were assessed to confirm successful establishment of the PCOS model. Subsequently, the PCOS mice were randomly divided into four groups (n=8): PCOS model group, low-dose laurolitsine group (LL-L group, 50 mg/kg/d), high-dose laurolitsine group (LL-H group, 100 mg/kg/d), and metformin group (Met group, 200 mg/kg/d) (19). The normal group continued to receive a basic diet, while the PCOS model group, LL-L group, LL-H group, and Met group continued with HFD. After the week 9, all animals were fasted for 8 h, blood samples were collected. Serum was centrifuged for analysis, and tissue samples were obtained for further experiments.

### 2.3 Estrous cycle determination

Vaginal smears were used to assess the estrous cycle of the mice (20). The vagina was flushed two to three times with 25 mL of sterile saline, and the vaginal fluid was then collected and placed on a slide. After air-drying at room temperature, slides were stained with methylene blue (MB). Vaginal cytology was used to assess the estrous cycle’s stage.

### 2.4 Oral glucose tolerance test

After 8 h of fasting, the blood glucose levels of the mice were measured, and the oral glucose dose was set at 2 g/kg body weight. Blood glucose levels were measured by drawing blood from the tail vein at 15, 60, 90, and 120 min after glucose administration (21).

### 2.5 Insulin tolerance test

After 8 h of fasting, the body weight of the mice was measured, and blood glucose levels were tested before the injection. The mice received an intraperitoneal injection of insulin at a dose of 0.5 U/kg body weight. Blood glucose levels were collected from the tail at 30, 60, 90, and 120 min after the injection.

### 2.6 Serum biochemical analysis

Blood samples were collected from the mouse’s eyeball and allowed to stand at room temperature for 4 h. Subsequently, the samples were centrifuged at 3500 rpm for 15 min at 4°C, and the serum was transferred to new centrifuge tubes for further analysis. Serum TG, CHO, LDL-c, HDL-c, ALT, and AST levels were measured according to the instructions of commercial assay kits. Additionally, serum levels of T, E2, LH, and FSH were determined using ELISA kits.

### 2.7 Hematoxylin and eosin staining

Fresh tissues were fixed in 4% formaldehyde at room temperature for 24 h. After fixation, the tissues were dehydrated through a graded ethanol series. Subsequently, the tissues were infiltrated with paraffin at 65°C, embedded, and sectioned into 4 μm-thick slices. The sections were floated in a 40°C water bath, placed on glass slides, and baked in a 60°C oven for adherence. Subsequently, the sections were deparaffinized in xylene three times and rehydrated through 100% and 95% ethanol for 5 min each. The sections were stained with hematoxylin for 5 min, rinsed for 10 min, differentiated in 0.7% hydrochloric acid ethanol for 10 s, and returned to blue with tap water for 10 min. The sections were then stained with eosin for 5 min, dehydrated with 95% and absolute ethanol for 5 min each, cleared twice in xylene, and finally mounted with neutral resin (22).

### 2.8 RNA-Seq analysis

Ovaries were collected from three mice in each of Normal, PCOS and LL (100 mg/kg) groups for analysis. Total RNA was extracted and its purity and integrity were verified using agarose gel electrophoresis, a Qubit 4.0 fluorometer/MD, and a Q sep 400 bioanalyzer. A cDNA library was constructed, and its quality was evaluated using an Agilent Bioanalyzer 2100 system. The library was sequenced on the Illumina NovaSeq platform, generating 150 bp paired-end reads. Differentially expressed genes (DEGs) were identified and analyzed using SangerBOX (http://vip.sangerbox.com), along with Gene Ontology (GO) and Kyoto Encyclopedia of Genes and Genomes (KEGG) enrichment analyses.

### 2.9 16S rDNA sequencing

Fresh mouse fecal samples were collected, and total DNA was extracted using a commercial kit from Wetware (Wuhan, China). The V3-V4 region of 16S rDNA as then amplified using specific primers. Upstream primers with barcode tags were employed to distinguish different samples within the same library. The TruSeq Nano DNA LT sample preparation kit was used for library construction, and sequencing was performed on the Illumina MiSeq PE300 platform. Data quality control and analysis were conducted using the QIIME2 platform.

### 2.10 Data analysis

Statistical analyses were conducted using GraphPad Prism 8 (GraphPad Software Inc., La Jolla, CA). The normality of data distribution was assessed using the Shapiro-Wilk test and the Kolmogorov-Smirnov (K-S) test. All data included in the statistical analyses were confirmed to follow a normal distribution. For data that followed a normal distribution, one-way analysis of variance (ANOVA) was utilized to compare differences between groups. Results were presented as mean ± standard deviation (± SD). Differences were considered statistically significant at *P*< 0.05.

## 3 Results

### 3.1 DHEA plus HFD induces PCOS in mice

**Figure 1:**
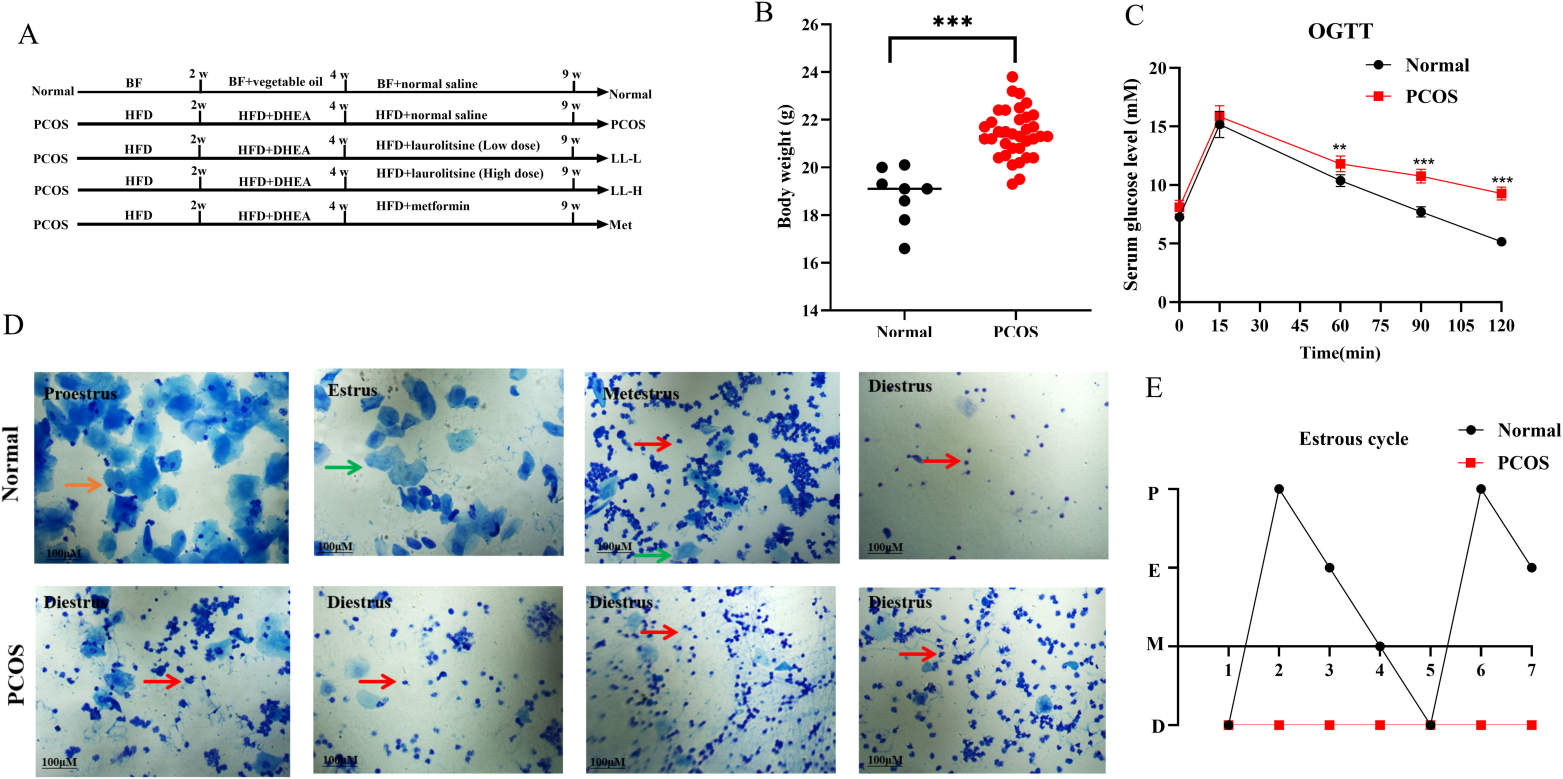
DHEA plus HFD induces PCOS in mice. **(A)** A schematic representation of the animal experiment. **(B)** Changes in body weight between normal and PCOS mice. **(C)** Glucose tolerance levels between normal and PCOS mice. **(D)** Vaginal smears from different stages of the estrous cycle between normal and PCOS mice. Red arrows indicating nuclear epithelial cells. Blue arrows indicating cornified squamous epithelial cells. Green arrows indicating leukocytes. **(E)** Trends in the estrous cycle. Data are presented as mean ± SD (Normal group, n = 8; PCOS group, n = 32). **p < 0.01, ***p < 0.001 *vs.* Normal.

To establish a PCOS mouse model, we used a combination of DHEA plus HFD (**Figure 1A**) (23). Compared with the normal group, the model mice exhibited significantly increased body weight and impaired glucose tolerance, as demonstrated by the OGTT results (**Figures 1B, C**), indicating severe glucose intolerance. Additionally, the estrous cycle of the DHEA plus HFD-induced mice were disrupted (**Figures 1D, E**). These results collectively confirm the successful establishment of the PCOS mouse model.

### 3.2 LL successfully improves glucose-lipid metabolism and insulin resistance in PCOS mice

**Figure 2:**
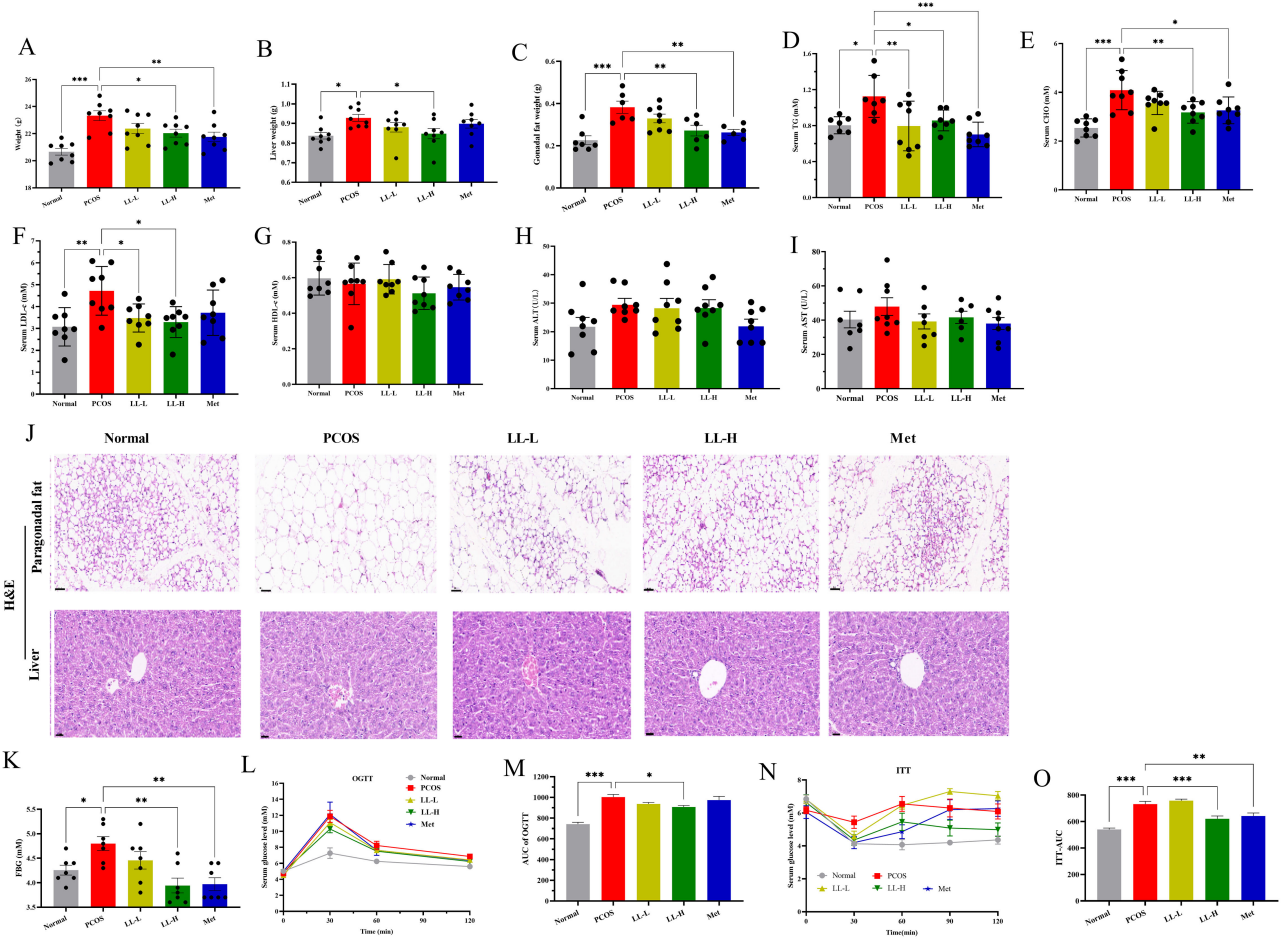
LL successfully improves glucose-lipid metabolism and insulin resistance in PCOS mice. **(A)** Body weight. **(B)** Liver weight. **(C)** Gonadal fat weight. **(D)** Serum triglyceride (TG) levels. **(E)** Serum total cholesterol (CHO) levels. **(F)** Serum low-density lipoprotein cholesterol (LDL-c) levels. **(G)** Serum high-density lipoprotein cholesterol (HDL-c) levels. **(H)** Serum alanine aminotransferase (ALT) levels. **(I)** Serum aspartate aminotransferase (AST) levels. **(J)** H&E staining of liver and gonadal fat tissues. **(K)** Fasting blood glucose levels. **(L, M)** Oral glucose tolerance test (OGTT). **(N, O)** Insulin tolerance test (ITT). Data are presented as mean ± SD (n=8 per group). *p < 0.05, **p < 0.01, ***p < 0.001.

To evaluate the effects of LL on PCOS, we administered LL intervention in a PCOS mouse model induced by DHEA combined with HFD. Compared with the normal group, the body weight, liver weight, and gonadal fat weight of PCOS mice were significantly increased (**Figures 2A–C**). However, after oral administration of LL (100 mg/kg/d), the body weight, liver weight, and gonadal fat weight of PCOS mice were significantly reduced (**Figures 2A–C**). Furthermore, the serum TG, CHO, and LDL-c levels were significantly elevated in PCOS mice (**Figures 2D–G**), but LL intervention significantly reduced these lipid parameters, indicating a notable improvement in lipid metabolism. Serum ALT and AST levels showed no significant changes (**Figures 2H, I**), and both LL-L and LL-H did not exhibit hepatotoxicity (**Figure 2J**), demonstrating a favorable safety profile. Adipose tissue is considered a key factor in the etiology of PCOS, particularly in metabolic abnormalities associated with overweight and obesity (24). H&E staining revealed marked accumulation of gonadal fat in PCOS mice, accompanied by more numerous and larger lipid droplets (**Figure 2J**). LL intervention significantly reduced the size of lipid droplets. Furthermore, PCOS mice exhibited significantly elevated fasting blood glucose (FBG), impaired glucose tolerance, and increased insulin resistance (**Figures 2K–O**). After LL treatment, the FBG levels, glucose tolerance, and insulin resistance were significantly improved in PCOS mice. Collectively, these findings indicate that LL significantly alleviates lipid metabolism disorders, impaired glucose tolerance, and insulin resistance induced by DHEA plus HFD in PCOS mice.

### 3.3 LL alleviates hormonal imbalance in PCOS mice

**Figure 3:**
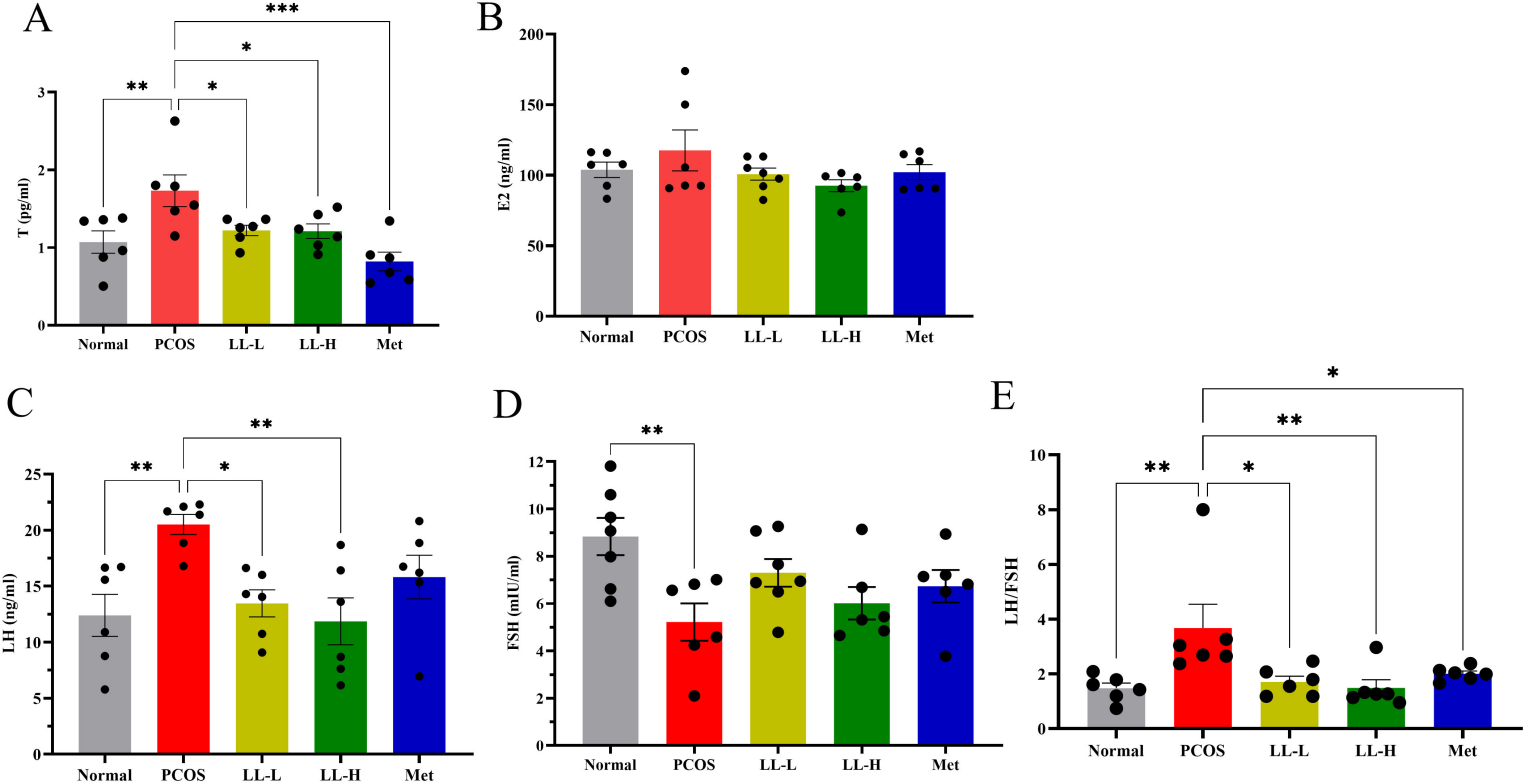
LL alleviates hormonal imbalance in PCOS mice. **(A)** Serum testosterone (T) levels. **(B)** Serum estradiol (E2) levels; **(C)** Serum luteinizing hormone (LH) levels; **(D)** Serum follicle-stimulating hormone (FSH) levels. **(E)** LH/FSH ratio. Data are presented as means ± SD (n = 8 per group). *p < 0.05, **p < 0.01, ***p < 0.001.

Elevated androgen levels, decreased ovulation, and polycystic ovarian morphologic alterations are major characteristics of PCOS in patients, which are similarly observed in the PCOS mice model. We evaluated serum FSH, LH, E2, and T levels to assess the effects of LL on hormonal regulation. Compared with the normal group, serum T and LH levels were significantly increased, while FSH levels were significantly decreased in PCOS mice. No significant change was observed in E2 levels (**Figures 3A–D**). Following LL intervention, serum T and LH levels in PCOS mice were significantly decreased, similar to the effects observed Met group (**Figure 3A, C**). In addition, both LL and Met dramatically reduced the LH/FSH ratio in PCOS mice (**Figure 3E**). These results indicate that LL effectively alleviates hormonal imbalance, thereby improving PCOS-like features in mice.

### 3.4 LL improves ovarian function in PCOS mice

**Figure 4:**
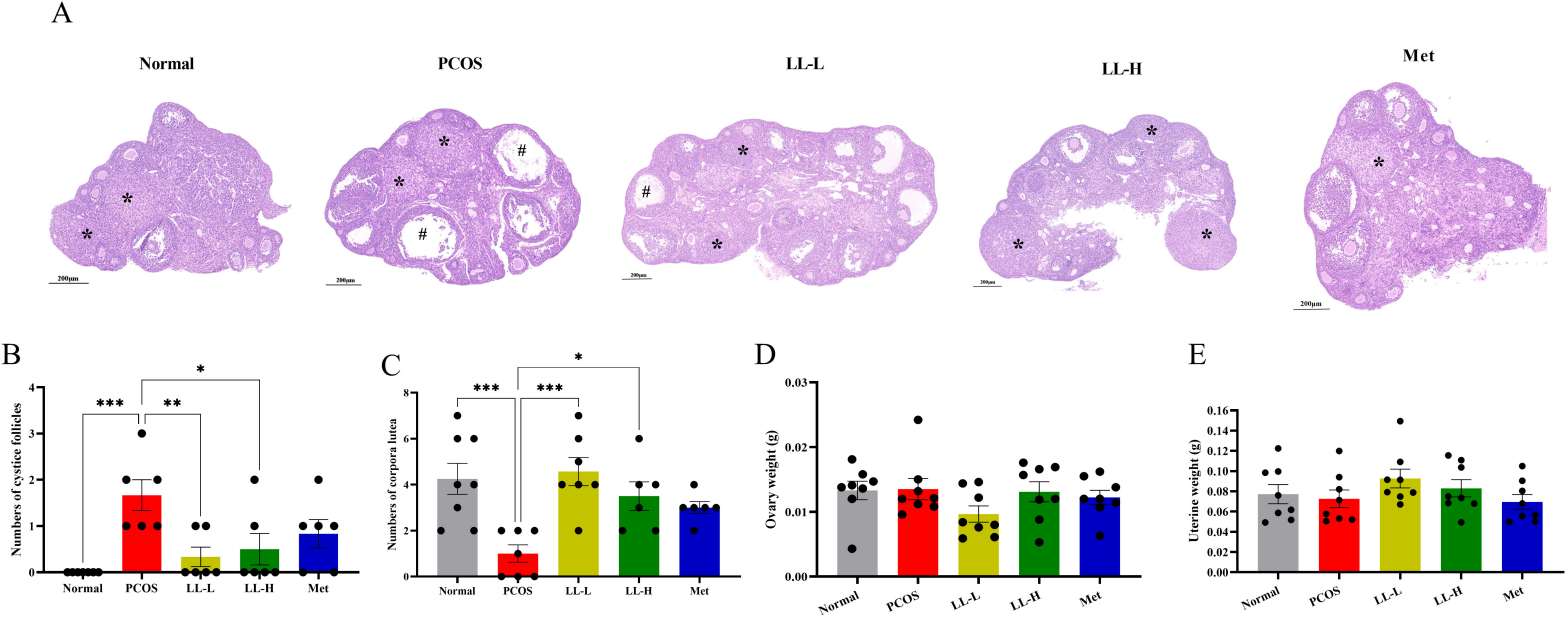
LL improves ovarian pathologic damage in PCOS mice. **(A)** Pathological changes in ovary morphology. **(B)** Number of cystic follicles. **(C)** Number of corpora lutea. **(D)** Ovary weight. **(E)** Uterus weight. Data are presented as mean ± SD (n = 8 per group). *p < 0.05, **p < 0.01, ***p < 0.001.

Ovarian dysfunction is a key characteristic of PCOS. To confirm the ameliorative effects of LL on folliculogenesis and ovulation, we assessed ovarian morphology in PCOS mice. Compared to normal mice, PCOS mice showed a reduced number of corpora lutea and a significantly increased number of follicles, accompanied by vacuolization and structural disorganization (**Figures 4A–C**). After LL intervention, the formation of corpora lutea increased, and the number of follicles with vacuolated cystic dilatation decreased in PCOS mice (**Figures 4A–C**). However, LL did not significantly affect ovary weight or uterus weight in PCOS mice (**Figures 4D, E**). These results suggest that LL may improve ovarian function in PCOS mice.

### 3.5 RNA-seq predicts the mechanisms of action of LL in PCOS mice

**Figure 5:**
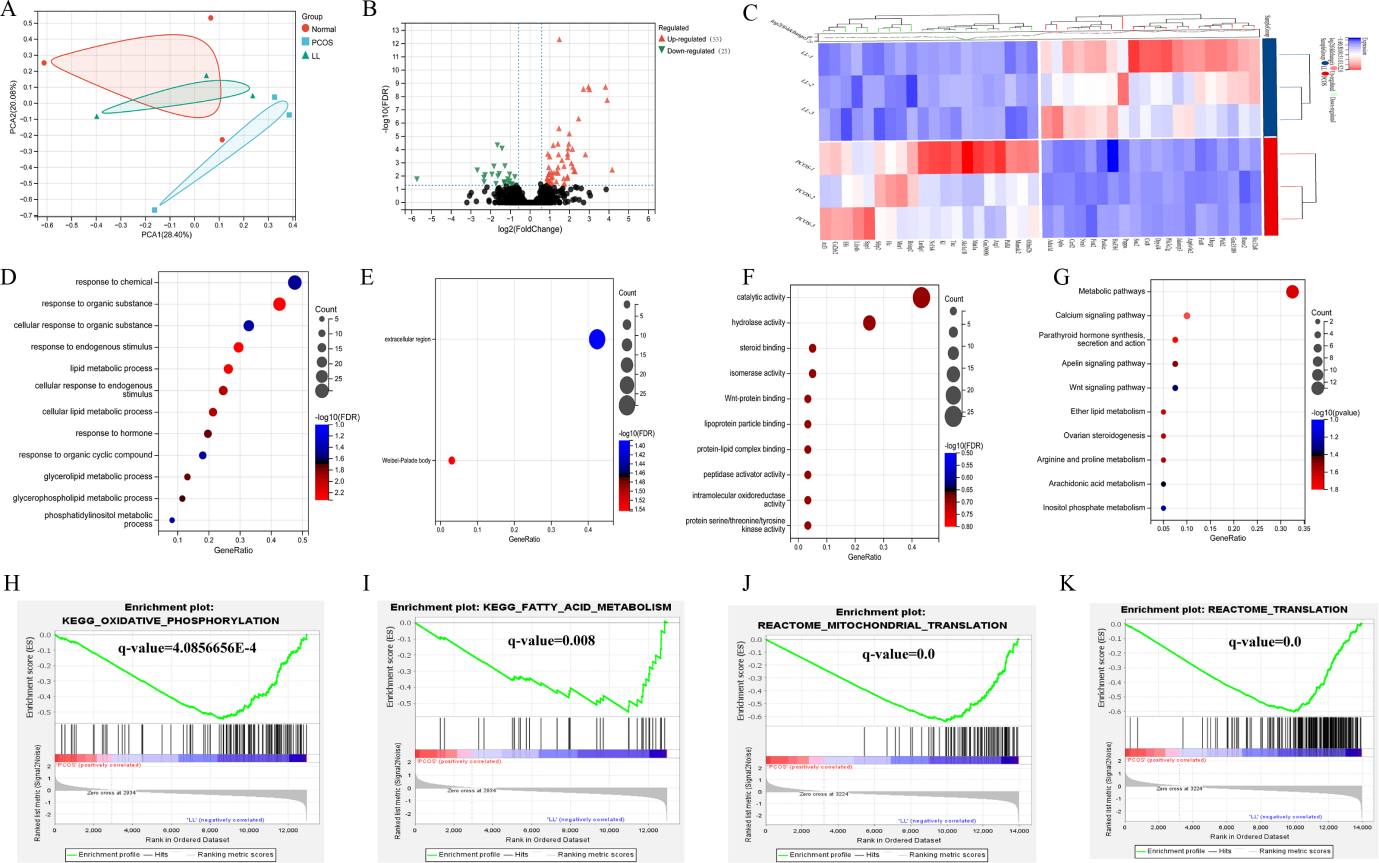
RNA-seq predicts the mechanisms of action of LL in PCOS. **(A)** Principal component analysis (PCA). **(B)** Volcano plots of differentially expressed genes (DEGs). **(C)** Heatmap of DEGs. **(D)** Gene Ontology (GO) analysis for biological processes, **(E)** cellular components, and **(F)** molecular functions. **(G)** Kyoto Encyclopedia of Genes and Genomes (KEGG) pathway enrichment analysis. **(H–K)** The Gene Set Enrichment Analysis (GSEA) analysis.

To further explore the potential mechanisms of LL’s effects in PCOS mice, we conducted RNA-seq analysis on ovary tissues from PCOS mice with or without LL treatment. Principal component analysis (PCA) showed a clear separation between the PCOS group and the LL (100mg/Kg/d) group (**Figure 5A**). The volcano plot revealed 53 upregulated and 25 downregulated genes in the LL group compared with the PCOS group (**Figure 5B**). A heatmap illustrated differential expression of these genes (**Figure 5C**). Among them, we observed significant modulation of Lhcgr (luteinizing hormone/choriogonadotropin receptor) and Tnc (tenascin-c), both of which are involved in critical ovarian function. Lhcgr is essential for LH-mediated follicular maturation and ovulation; its downregulation in PCOS may lead to impaired follicle maturation and anovulation, while LL treatment restored Lhcgr expression, suggesting a potential mechanism for LL in improving ovarian function (25, 26). In contrast, Tnc known to mediate tissue remodeling and inflammation, was upregulated in PCOS mice, reflecting excessive extracellular matrix deposition and granulosa cell apoptosis (27). LL treatment downregulated Tnc, suggesting that LL may alleviate ovarian inflammation and fibrosis, promoting healthier follicular development. GO analysis revealed that LL treatment primarily impacted biological processes such as response to organic substances, hormones and endogenous stimuli (**Figures 5D–F**). These findings are consistent with the potential therapeutic effects of LL on ovarian function and metabolic health in PCOS. Additionally, KEGG pathway enrichment analysis indicated that LL primarily affected pathways associated with ovarian steroidogenesis, apoptosis signaling, ether lipid metabolism, and arginine and proline metabolism (**Figure 5G**). These pathways are critical for maintaining ovarian function and metabolic homeostasis, further supporting LL’s role in improving ovarian function and metabolic regulation. Further analysis through Gene Set Enrichment Analysis (GSEA) demonstrated that oxidative phosphorylation, fatty acid metabolism, mitochondrial translation, and gene groups related to translation were significantly downregulated in the ovary tissues of PCOS mice, while LL reversed these genomic changes (**Figures 5H–K**). Notably, oxidative stress has been reported to play a critical role in hyperandrogenemia-induced organ dysfunction, including kidney injury, further supporting the involvement of oxidative stress pathways in PCOS pathogenesis (28). These results suggest that LL may improve PCOS by modulating key metabolic processes, including hormone response, fatty acid metabolism, and lipid metabolism in the ovary. The gene expression patterns after LL treatment are closely associated with the phenotypic improvements in ovarian function and metabolic regulation. Specifically, the upregulation of Lhcgr and downregulation of Tnc indicate that LL restores key signaling pathways involved in folliculogenesis and reduces ovarian inflammation. GO, KEGG, and GSEA analyses further support that LL exerts therapeutic effects by modulating pathways such as steroidogenesis, oxidative stress, and mitochondrial function, thereby improving ovarian function and alleviating metabolic dysregulation in PCOS mice.

### 3.6 LL effectively regulates PCOS gut microbiota

**Figure 6:**
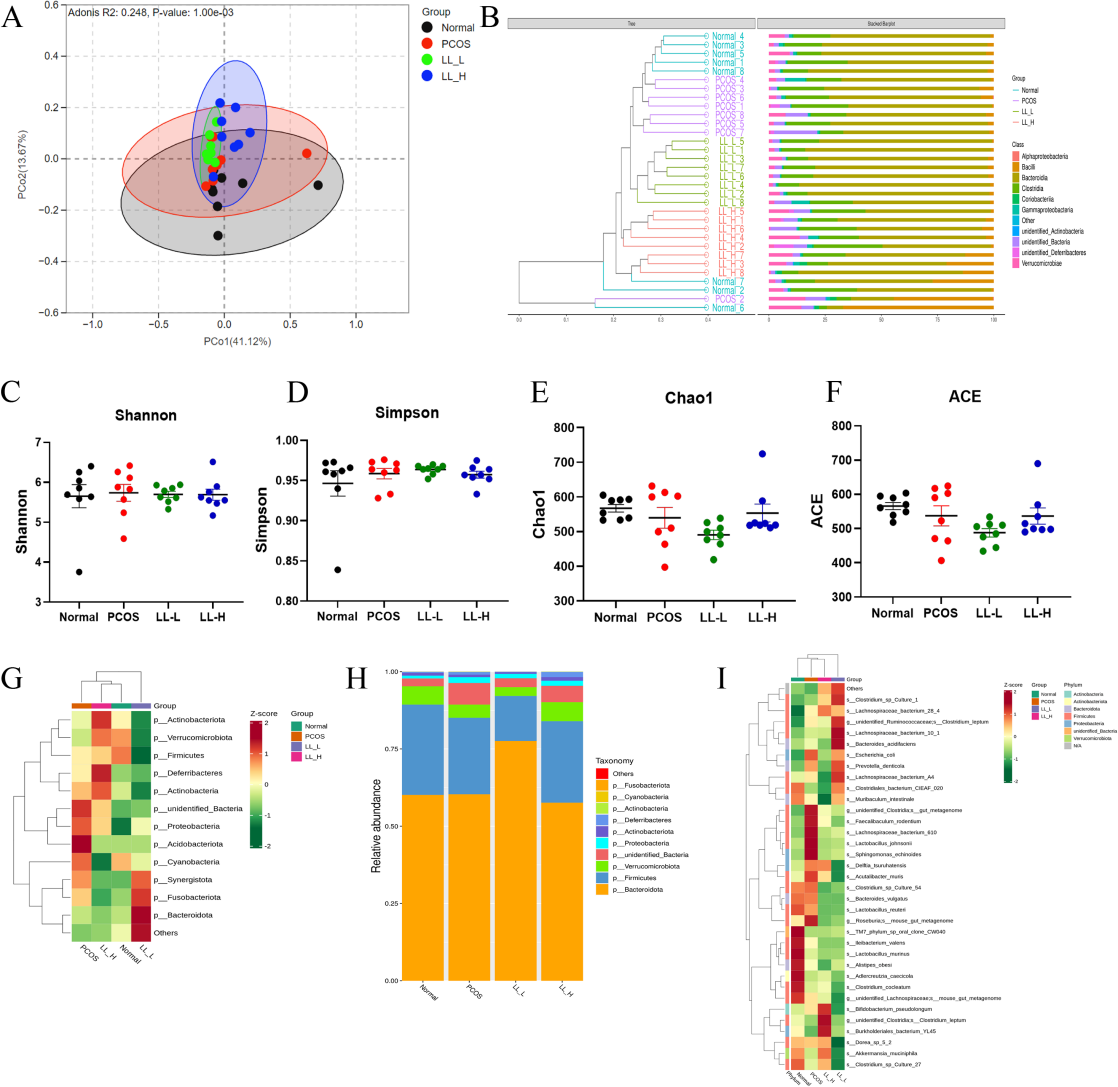
LL alters microbiota composition in PCOS mice. **(A)** The β-diversity based on the principal co-ordinates analysis (PCoA). **(B)** Unweighted Pair-group Method with Arithmetic Mean (UPGMA) clustering. The α-diversity of the gut microbiota analyzed using **(C)** Shannon index, **(D)** Simpson index, **(E)** chao1, **(F)** ACE. **(G)** Heatmap representing the changes in intestinal microbial at the phylum level. **(H)** Relative abundance of gut microbiota at a phylum level. **(I)** Heatmap representing the changes in intestinal microbiota at the species and genus levels.

The gut microbiota has been recognized as an essential mediator of metabolic diseases including PCOS, and modulation gut microbiota is considered an effective strategy for the prevention and treatment of these related diseases (29). α-diversity reflects within-sample richness and evenness, while β-diversity describes between-sample compositional differences. Through 16S rDNA sequencing analysis, principal co-ordinates analysis (PCoA) and unweighted pair-group method with arithmetic mean (UPGMA) showed that both LL-L and LL-H interventions distinctly shifted the gut microbiota composition away from that of the PCOS group (**Figures 6A, B**). However, α-diversity analyses (Shannon, Simpson, Chao1, and ACE) did not reveal significant differences in species richness and diversity among the groups (**Figures 6C–F**), indicating that LL intervention primarily affects microbial composition rather than overall diversity.

At the phylum level, *Proteobacteria (or Pseudomonadota)* was significantly elevated in PCOS mice. This phylum is closely associated with microbial dysbiosis and low-grade inflammation driven by endotoxins (30), a chronic inflammatory state that has been identified as a key contributor to insulin resistance and hyperandrogenism in PCOS. Notably, LL-L and LL-H significantly reduced the abundance of *Proteobacteria*, which may help alleviate gut-derived inflammation and systemic metabolic stress (**Figures 6G, H**).

At the species level, 29 species were found to be significantly altered between the PCOS group and LL-treated groups (**Figure 6I**). Specifically, *Faecalibaculum rodentium*, *Lactobacillus johnsonii*, *Bacteroides vulgatus*, and *Sphingomonas echinoides* were markedly enriched in PCOS mice. These species have been linked to low-grade intestinal inflammation, gut microbiota dysbiosis, and disrupted estrogen metabolism, all of which are central to PCOS pathogenesis (31, 32). Notably, *Lactobacillus johnsonii* is known for its high β-glucuronidase activity, which may affect estrogen recycling and exacerbate hormonal imbalance (33). LL treatment markedly decreased the abundance of these species, thereby helping to restore gut microbial balance. Meanwhile, LL intervention, particularly LL-H promoted the enrichment of beneficial species such as *Akkermansia muciniphila*, which has been shown to be a beneficial bacterium associated with intestinal mucosal barrier integrity and enhanced anti-inflammatory responses. It plays a positive role in improving obesity-related metabolic syndromes and regulating glucose and lipid metabolism (34). These findings suggest that LL modulates the gut microbiota in a dose-dependent manner, targeting key species involved in metabolic regulation and hormonal balance. This remodeling of the gut microbial community may underlie the potential therapeutic effects of LL in alleviating PCOS-related phenotypes.

**Figure 7:**
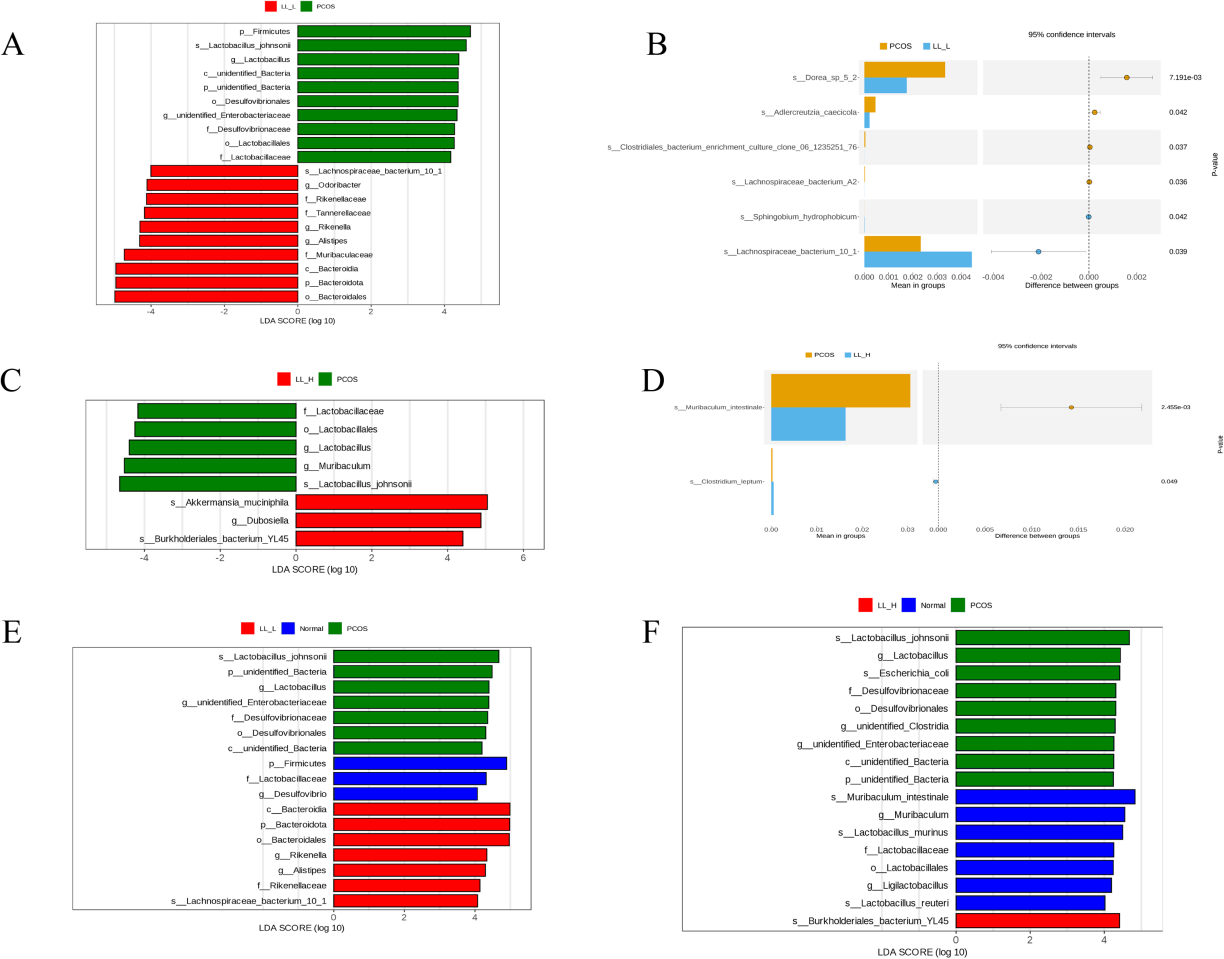
**(A, C, E, F)** Linear discrimination analysis (LDA) effect size LEfSe. Histogram of the LDA scores computed for differentially abundant species among the Normal, PCOS, LL-L, LL-H. The LDA scores (log 10) > 2 are listed. **(B, D)** T test.

Through T-test and LEfSe analysis, we further investigated the changesn gut microbiota following LL intervention. In the PCOS group, *Lactobacillus johnsonii* was dominant species. In contrast, *Lachnospiraceae bacterium 10_1* dominated in the LL-L group (**Figures 7A, B, E**). In the LL-H group, *Akkermansia muciniphila* and *Burkholderiales bacterium YL45* became the dominant species, while *Lactobacillus johnsonii* remained the dominant species in the PCOS group (**Figures 7C, D, F**). Notably, *Lactobacillus johnsonii*, enriched in the PCOS group, has been linked to intestinal inflammation and hormonal disruption, potentially contributing to the pathogenesis of PCOS. After LL intervention, the abundance of *Lactobacillus johnsonii* decreased, while the beneficial bacterium *Akkermansia muciniphila* increased, which are associated with anti-inflammatory effects and improved metabolic health. These findings further support the gut microbiota remodeling effect of LL intervention.

**Figure 8:**
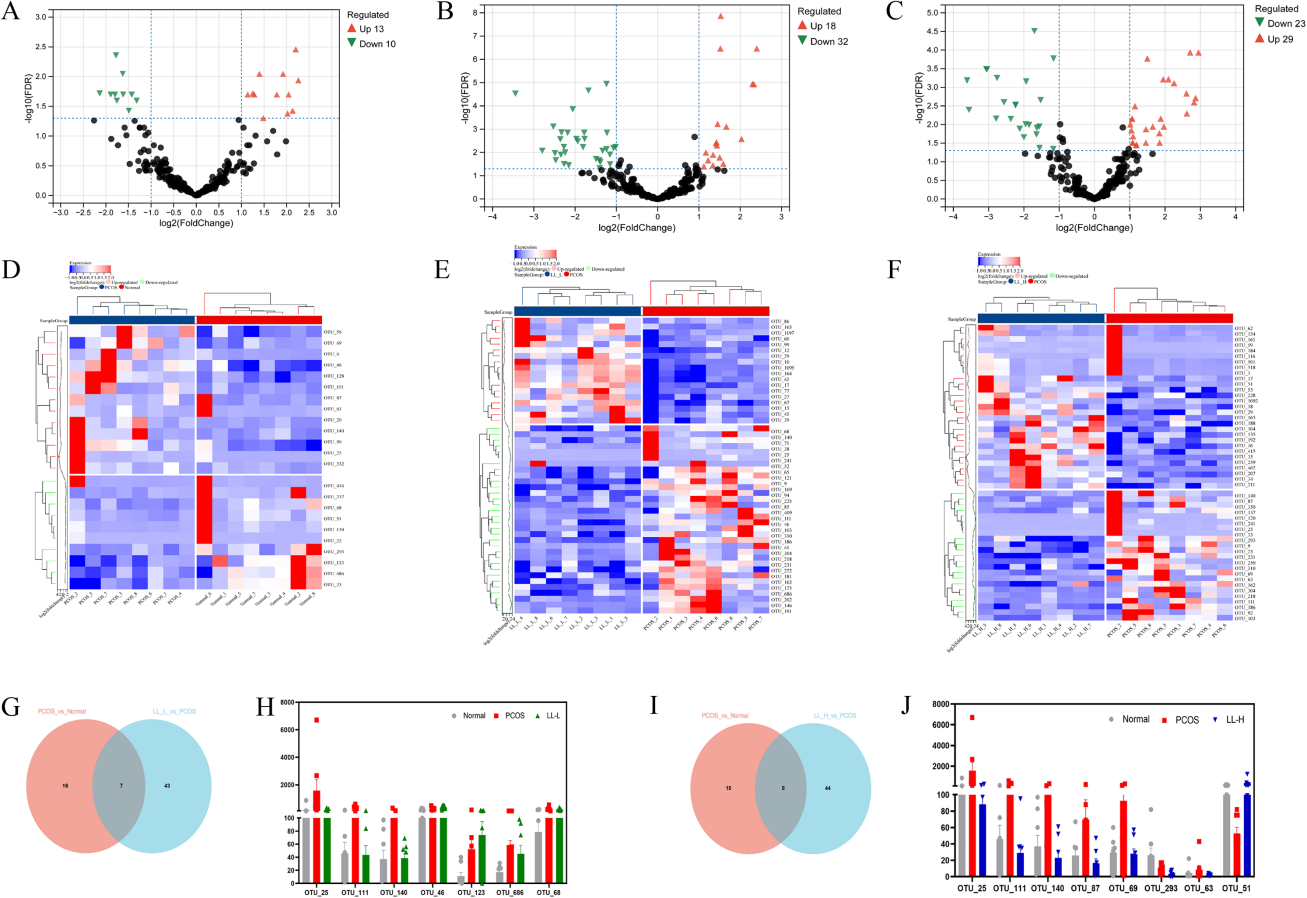
LL intervention modulates gut microbial OTU composition in PCOS mice. **(A–C)** Volcano plots showing differential expressed OTUs between groups analyzed using DESeq2. **(A)** PCOS group *vs.* normal group. **(B)** LL-L group *vs.* PCOS group. **(C)** LL-H group *vs.* PCOS group. **(D–F)** Heatmaps of the differentially expressed OTUs. **(G, I)** Venn diagrams of differentially regulated OTUs in comparisons between the PCOS group and each treatment group. **(H, J)** Histogram of different OTU expressions.

OTU (Operational Taxonomic Unit) is a term commonly used in microbial ecology to classify groups of closely related individuals based on DNA sequence similarity, typically in 16S rRNA gene sequencing analysis. Further analysis of OTU changes using DESeq2 revealed that, compared to the normal group, 13 bacterial OTUs were upregulated and 10 were downregulated in the PCOS group (**Figures 8A, D**). In the LL-L group, 18 bacterial OTUs were upregulated and 32 were downregulated compared to the PCOS group (**Figures 8B, E**), while in the LL-H group, 29 OTUs were upregulated and 23 were downregulated (**Figures 8C, F**). Venn diagram analysis further demonstrated that LL significantly regulated OTUs associated with PCOS. Specifically, LL-L treatment significantly downregulated OTUs such as OTU_25, OTU_111, OTU_140, and OTU_686, while LL-H treatment reduced OTUs including OTU_25, OTU_111, OTU_140, OTU_87, and OTU_69, while upregulating OTU_51 (**Figures 8G–J**). These findings indicated that LL significantly altered gut microbiota at the OTUs levels, educing PCOS-related pathogenic taxa and enriching beneficial microbial populations, thereby contributing to the improvement of gut dysbiosis in PCOS mice. LL treatment may specifically modulate the gut microbiota composition in the PCOS model, particularly by increasing the abundance of *Akkermansia muciniphila*, while reducing the relative abundance of *Proteobacteria* and *Lactobacillus johnsonii*. These changes may help improve the host’s metabolic function, inflammatory responses, and endocrine balance, thereby offering a potential microbiota-targeted intervention strategy for the treatment of PCOS.

## 4 Discussion

PCOS is a metabolic syndrome commonly associated with insulin resistance, ovarian dysfunction, and gut microbiota dysbiosis (35, 36). However, most clinical treatments fail to effectively alleviate PCOS symptoms. Increasing evidence suggests that traditional Chinese medicine can effectively treat PCOS in both animal and human trials (37). LL is a natural alkaloid in traditional Chinese medicine, previously shown to improve diabetic db/db mice (19). Building on this, we assessed the effects and probable mechanisms of LL on the PCOS mice model induced by DHEA plus HFD. Our research demonstrated that LL significantly reduced dyslipidemia and insulin resistance, improved hormone levels, and alleviated ovarian dysfunction. These effects may be mediated through processes such as response to hormone, fatty acid metabolism, lipid metabolism, regulation of gut barrier function, alleviation of gut dysbiosis, and enhancement of beneficial gut microbiota. These findings suggest that LL may play an essential role in the treatment of PCOS.

Obesity and dyslipidemia are closely associated with PCOS, and both significantly impact the fertility of PCOS patients. Therefore, weight management is a critical clinical objective in the symptomatic treatment of PCOS (38). In our study, we found that LL intervention effectively reduced gonadal fat accumulation and mitigated body weight gain in PCOS mice. Meanwhile, dyslipidemia, characterized by elevated levels of TG, CHO, and LDL-c, is the most common metabolic abnormality observed in PCOS patients (39). LL intervention significantly reduced serum LDL-c, CHO, and TG levels in PCOS mice, demonstrating its capacity to improve lipid metabolism and providing an additional therapeutic benefit for managing PCOS-related metabolic disturbances (40). Furthermore, LL could significantly improve the FBG levels, glucose tolerance, and insulin resistance of PCOS mice. Notably, LL-H (100 mg/kg) demonstrated pronounced improvements in key parameters, including body weight, liver and gonadal fat weight, serum lipid levels, and glucose tolerance, highlighting a dose-dependent efficacy of LL in alleviating PCOS symptoms. These findings indicate that LL significantly alleviates lipid metabolism disorders, impaired glucose tolerance, and insulin resistance induced by DHEA plus HFD in PCOS mice.

Hyperandrogenism is one of the main causes and contributing factors to the development of PCOS, and it is a characteristic manifestation of hormonal imbalance in the disease. In PCOS patients, excessive LH suppresses FSH, disrupting endocrine balance and impairing follicular development, leading to an increased LH/FSH ratio (41). Additionally, elevated T levels affect metabolism and appetite, resulting in metabolic imbalance and weight gain (42). Therefore, treating PCOS requires lowering blood androgen levels and addressing hormonal imbalance (43). Our research demonstrated that LL significantly improved hormonal imbalance in PCOS mice by reducing the serum LH/FSH ratio and testosterone levels.

In this study, we found that LL intervention effectively improves the gut microbiota structure in PCOS model mice. Although there were no significant changes in α-diversity, β-diversity analysis indicated that LL reshaped the microbiota composition, making it closer to a normal state, suggesting its beneficial regulatory effect on the gut microbiome. This is consistent with previous studies indicating that the pathology of PCOS does not significantly impact the overall diversity of the gut microbiota, but there are differences in microbial composition (44). Regarding microbiota composition, *Proteobacteria* and *Lactobacillus johnsonii* were significantly increased in PCOS mice, which is closely related to the inflammatory state. LL-L and LL-H intervention reduced its abundance, potentially helping to alleviate systemic inflammation. LL-H significantly enriched *Akkermansia muciniphila*, a bacterium known to help strengthen the gut barrier and improve metabolic disorders (34). This suggests that LL may improve PCOS-related endocrine and metabolic abnormalities by modulating key microbiota members.

Although LL showed promising therapeutic efficacy in our PCOS mouse model, further research-including antibiotic intervention and fecal microbiota transplantation experiments- is necessary to clarify the precise mechanisms underlying LL’s regulation of gut microbiota. In addition, long-term safety evaluations and clinical studies are required to fully understand its potential limitations or side effects.

## 5 Conclusion

Our study demonstrated that LL alleviates PCOS symptoms induced by DHEA plus HFD in mice, showing improvements in insulin resistance, hormonal disorder, and ovarian dysfunction. The potential mechanisms underlying LL’s ameliorative effects on PCOS may involve the regulation of ovarian hormone response and alterations of the gut microbiota. In summary, LL holds a promising therapeutic treatment for PCOS.

## Data Availability

The datasets generated for this study can be found in the NCBI database: PRJNA1307766.
